# Supplementary material for: Wearable bio-adhesive metal detector array (BioMDA) for spinal implants
Source: Nat Commun. 2024 Sep 6;15:7800. doi: 10.1038/s41467-024-51987-2 (PMC11379874; doi:10.1038/s41467-024-51987-2)
Supplement: Supplementary file 1 — Supplementary Information [file 41467_2024_51987_MOESM1_ESM.pdf]

Supplementary Materials for

Wearable Bio-adhesive Metal Detector Array (BioMDA) for Spinal Implants

Corresponding authors. emails: cgt20@mit.edu; xingeyu@cityu.edu.hk;

knan@zju.edu.cn; gshankar@mgh.harvard.edu; dengfli2-c@my.cityu.edu.hk

The file includes:

Supplementary Note1 to Note2

Supplementary Fig. 1 to Fig. 31

Legends for Supplementary Movie 1 to Movie 3

Other Supplementary Materials for this manuscript include the following:

Supplementary Movie 1 to Movie 3

Supplementary Note 1

Model analysis and simplification.

When the metal implants move relative to a sensing unit in BioMDA, electromagnetic interactions arise and eventually produce inducing signals from the coils embedded in the sensor array. The amplitude of the response signals reflects the kinematics of the permanent magnets and thus can be used to deduce the location of the implants. The electromagnetic interactions between implants and the sensing unit can be dissected into three steps: (1) as the implant approaches the permanent magnet, the implants are magnetized, exhibiting mutual attraction with corresponding permanent magnets. (2) the permanent magnets generate swing to the copper coils under the restriction of the holding film. (3) the changes in magnetic flux on coil surface generate inductive signals in the coil. Although the PET holding film restricts the kinematic response of the permanent magnet, the changing magnetic field as the permanent magnet swings back and forth produces a prominent electromotive force (emf) signal across the embedded coil. To analyze the electromagnetic-kinematic relationship between implants and the sensing unit, we assumed that the system can be decoupled into two relatively independent modules: the electromagnetic and kinematic models, and we solved this decoupling problem with theoretical and numerical methods.

In the electromagnetic model, we assumed that the magnet (radiance a and thickness h) was evenly magnetized with constant magnetization M, and the magnetic field density could be assumed gradient distribution horizontally (**Supplementary Fig.**

26). Thus, the magnet field B in the vacuum above the magnet could be modeled as a function of its vertical distance z as:

$$B = \frac{\mu_0 M}{2}\left[\frac{z}{\sqrt{z^2+a^2}} - \frac{z-h}{\sqrt{(z-h)^2+a^2}}\right] \tag{1}$$

Where μ_0 is the permeability of vacuum ($4\pi \times 10^{-7}$ H/m). Following Faraday's law of induction, the induced electromotive force ε across a horizontally placed circuit with N turns can be calculated as the rate of change of the total magnetic flux integrated over its area A with **Equation 2**:

$$\varepsilon_0(z) = -\frac{d}{dt}\int_{\Sigma} B\, dA = -\frac{\mu_0 NMAa^2}{2}\left[(z^2+a^2)^{-1.5} - ((z-h)^2+a^2)^{-1.5}\right]\frac{dz}{dt} \tag{2}$$

$$\varepsilon(z) = e^{-\gamma z}\varepsilon_0(z) = e^{-\gamma z}\xi_B(z)\dot{z} \tag{3}$$

For simplicity, the inducing signal could be treated as the product of three parts: attenuation $e^{-\gamma z}$, the term related to magnet field change $\xi_B(z)$, and velocity $\dot{z}$ (**Equation 3**). While ε_0 is the inducing signal in a vacuum, γ is the attenuation coefficient [1,2] of the propagation medium. The exponential decay term described how the amplitude of electromagnetic flux decreases as the wave propagates through the medium. Here, we neglected the change in phase during propagation. As it is used in the context of ultrasound and X-ray[3,4], the attenuation model is of a phenomenological expression. Although it is not easily supported by theory, it agrees well in practice and could be reasonably approximated by numerical methods.

Supplementary Note 2

Movement coupling between the CPS and the permanent magnet.

In practical applications, the electromagnetic induction caused by the moving implants z_i cannot be easily measured. Therefore, we took advantage of the coupling relationship between the implants and the magnet and developed a numerical method that approximated the scaling from the theoretical model ε_0 with the states of implants z_i to emf $\varepsilon(z_m)$ that the embedded coil acquired from the moving magnet z_m. Due to the restriction from PET holding film, the permanent generates a swing rotation in response to the external inductive force. Therefore, by taking the movement of magnet into consideration, the approximation process can be divided into two steps by decomposing the movement of the magnet into vertical movement and rotation.

In vertical direction, we assumed that the vertical movement of the implant z_i and magnet z_m share similar patterns. If the amplitude of z_m is proportional to z_i, we can switch the z_m in exponential and the derivative term in **Equation 5** to z_i by adding scale factors. With the design of BioMDA in **Fig. 2d**, we explored the potential kinematic space of the implant and observe that the magnet swings back and forth within amplitude of ±2 mm when an implant approaches and leaves the sensor with speed varying from 7.5 mm/s to 15 mm/s at locations of 2 mm - 6.5 mm underneath the sensor. For example, when an implant approaches the sensor from 15mm away to a minimum distance of 2 mm, the magnet swings up 2 mm. In such situation, letting $\xi(z) = (z^2 + a^2)^{-1.5} - ((z-h)^2 + a^2)^{-1.5}$, the value of $\xi(z_i)$ over $\xi(z_m)$ can be approximated with

an exponential function of z_i as in **Supplementary Fig. 27b**. Thus, we combine the attenuation function, scaling factors, and potential calibration residue for device differences into a form of exponential decay formula $sf(z_i) = a_1e^{-kz_i} + a_0$. As regression results indicates we further simplified the calibration function as $sf(z_i) = a_1e^{-z_i} + a_0$ setting $k = 1$. Because of the local minima z_0 around 2.834 mm (**Supplementary Fig. 27a**), when z_i is less than z_0, the peak emf happens when the implants pass the z_0 point instead of the point with minimum distance to senser unit. Thus, when z_i is less than z_0 we approximate the peak amplitude of $\xi_B(z_i)$ to $\xi_B(z_0)$ as in **Equation 5** to reduce error caused by the nonconvex details. For distance ranging from 1.25 mm to 6.5 mm and velocities ranging from 7 mm/s to 15.5 mm/s, the simulated $\varepsilon(z_m)$ as $sf_1(z_i)\xi_B(max(z_i, z_0))\dot{z}_i$ is plotted in **Supplementary Fig. 28a**.

As shown in **Supplementary Fig. 28a**, the differences between experiment data and the simulation exhibit a distortion along velocity axis. We posit that this distortion relates to the rotational primitives apart from vertical primitives. When the magnet swings back and forth in response to external electromagnetic interactions, the combined electromagnetic and gravitational forces cause the bending deformation in the holding film which in turn determines the rotating of the magnet filed. The rotation then affects electromagnetic induction across the coil and is reflected in the captured inducing signal $\varepsilon(z_m)$. We applied numerical methods to approximate the differences in **Supplementary Fig. 28a** and calibrated the experimental results and the theoretical values. The distortion in the velocity space was observed around 11.25 mm/s with

slopes following an exponential curve of z_i (**Supplementary Fig. 28b**), and the fitted results by considering both distance and velocity distortion is shown in **Supplementary Fig. 28c**. Here, we introduced a linear fit of z_i to compensate for the discrepancy in the center of the distortion.

By taking these into consideration, the scaling function and the amplitude of the inducing signal can be approximated with **Equation 4** and **Equation 5**, respectively:

$$SF(z,\dot{z}) = sf_1(z) + sf_2(z)(\dot{z} - (kz + b)) \tag{4}$$

$$\hat{\varepsilon}(z_m) \approx SF(z_i,\dot{z}_i)\xi_B(max(z_i,z_0))\dot{z}_i \tag{5}$$

where constant values k, b, and a_* in both calibration functions $sf_*(z) = a_1e^{-z} + a_0$ were estimated using regression and z_0 is the local minima of $\xi_B(z)$ around 2.834 mm.

Supplementary Figures

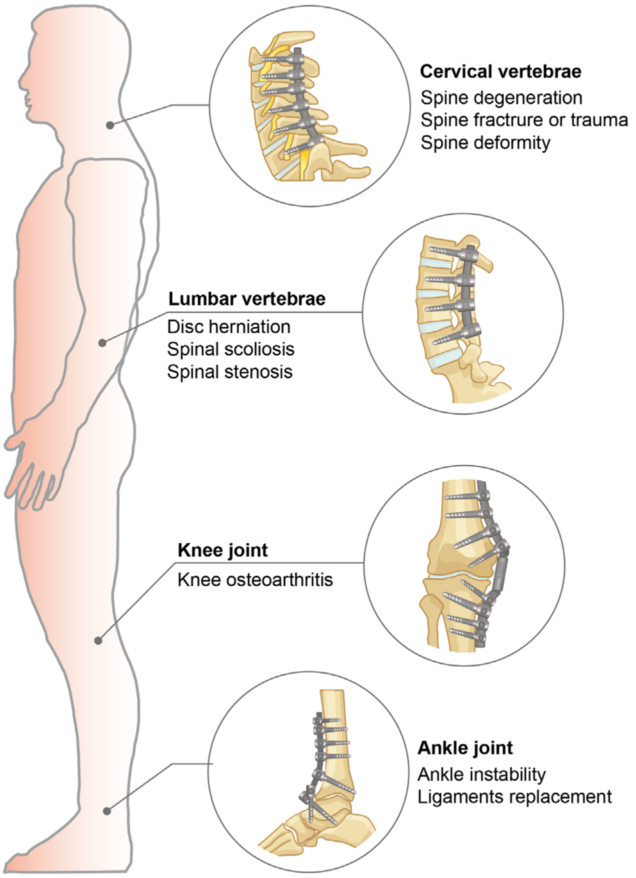

Supplementary Fig. 1 | Schematic illustration of BioMDA for diverse orthopedic implants localization.

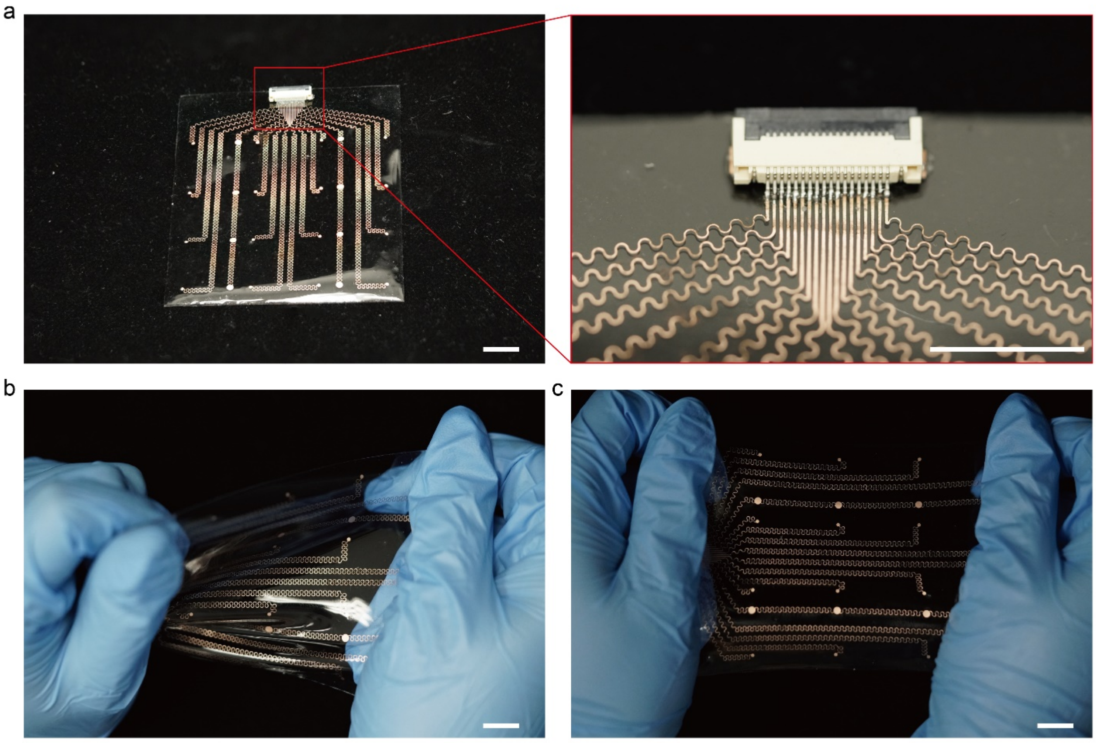

Supplementary Fig. 2 | Optical images of the flexible electrode of the BioMDA. a. Optical images of the electrode and enlarged view of the integrated connection port. Optical images of the flexible electrode undergoing twisting deformation (b) and stretching deformation (c). Scale bars: 1 cm.

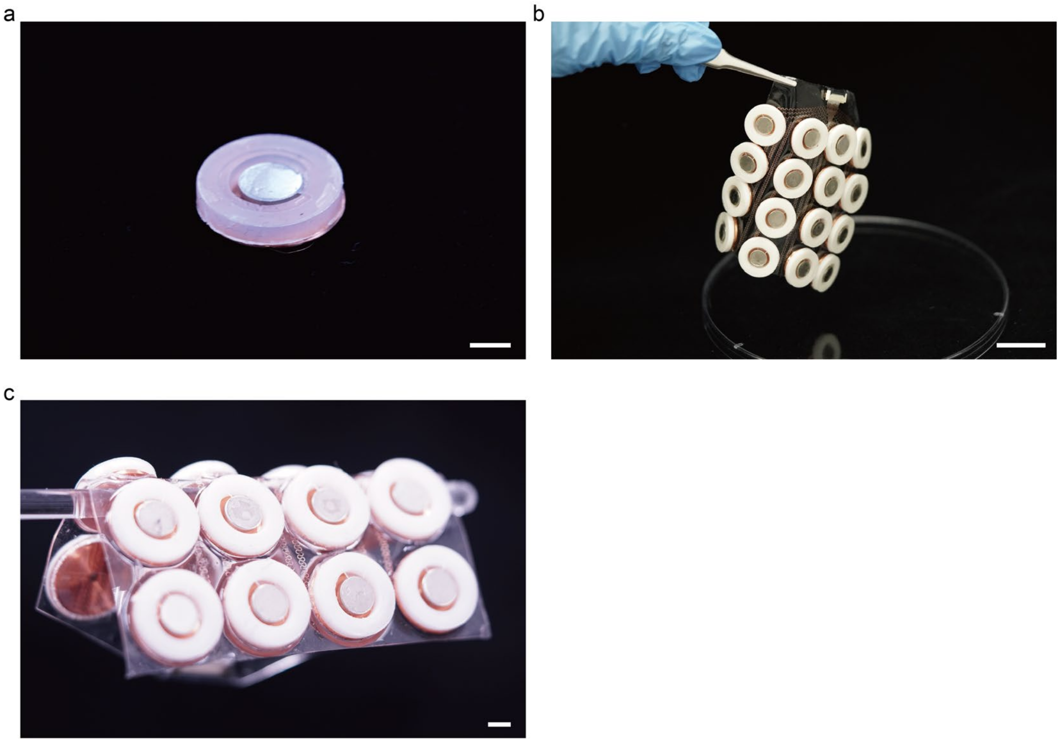

Supplementary Fig 3 | Optical images of the single sensing unit and assembled sensing units into a 4 × 4 sensing array. a. Optical image of the single sensing unit. b. Assembled sensing array. c. Assembled sensing array in bending state. Scale bars: 5 mm.

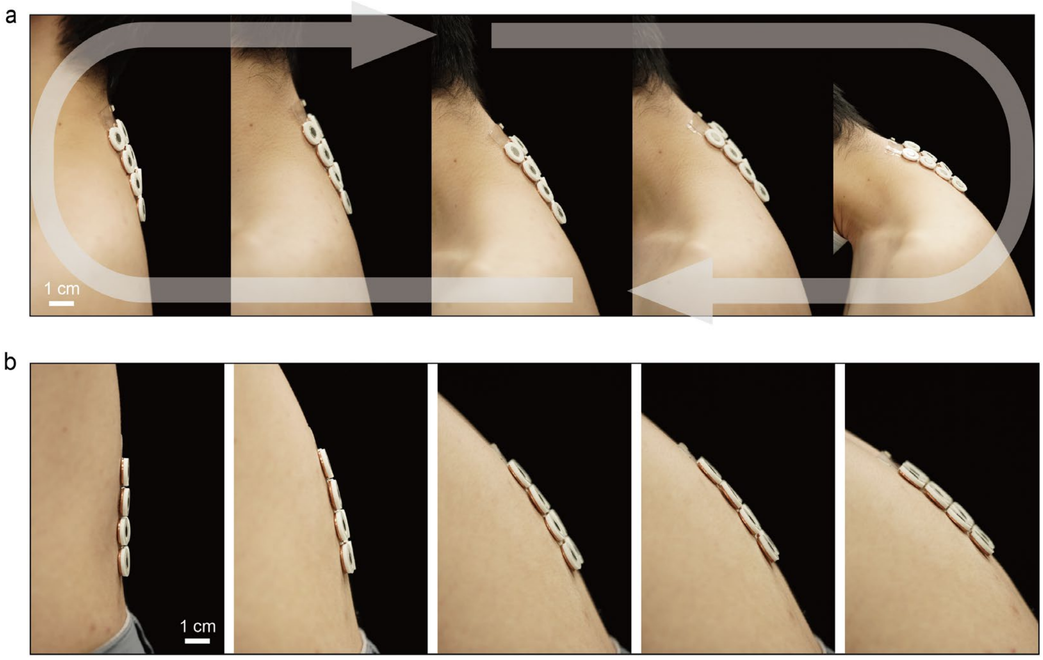

Supplementary Fig. 4 | Optical images showing the use process of BioMDA on cervical and lumbar spine. Optical images showing the ues process BioMDA mounting on user's neck (a) and back (b).

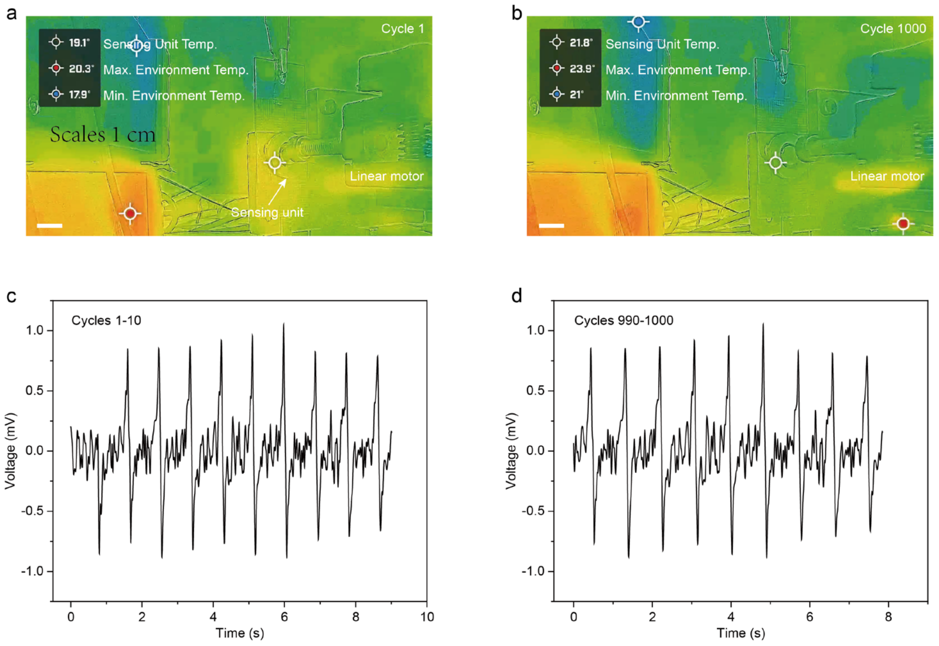

Supplementary Fig. 5 | Temperature stability of the sensing unit over 1000 cycles. a-b, Temperature and c-d, Sensing signals at cycle 1 and cycle 1000. Scale bars: 1 cm.

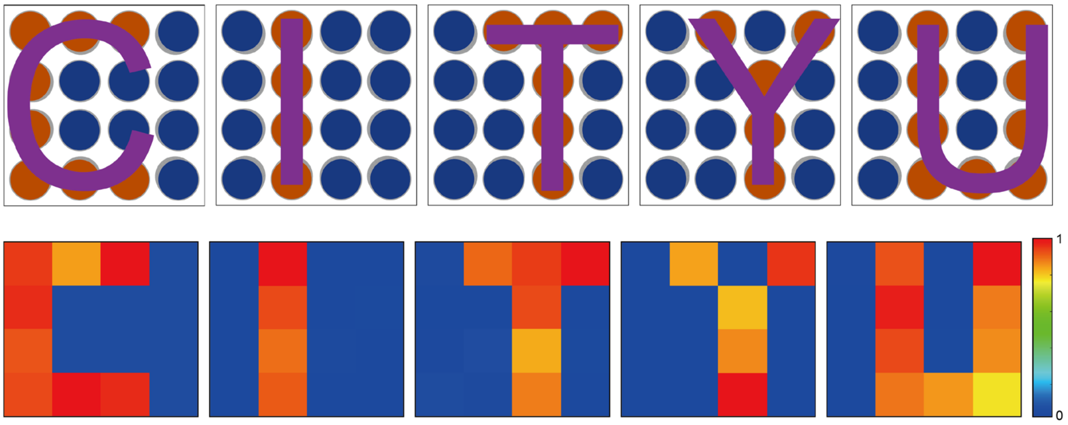

Supplementary Fig. 6 | Results showing the sensing uniformity and crosstalk suppression of the sensor array with a dynamic sensing pattern of “CITYU”.

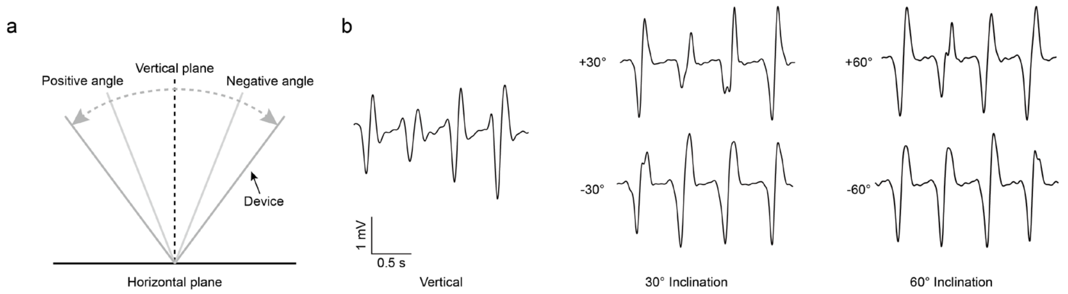

Supplementary Fig. 7 | Signal comparison on different device orientation. a. Schematic illustration on device orientation and device response signals (b).

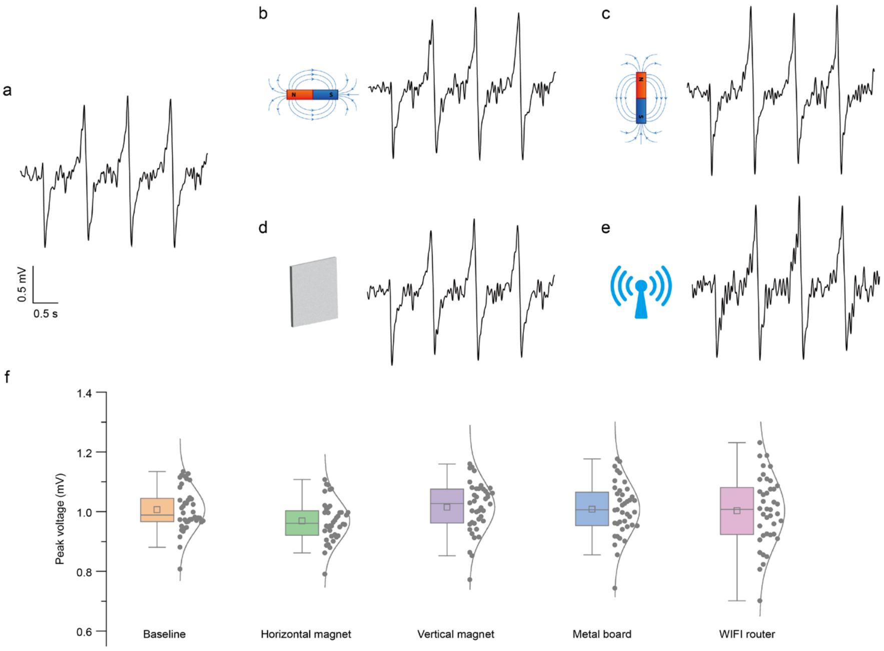

Supplementary Fig. 8 | Anti-interference capability evaluation of the device under different interference sources. Response signals without external interference (a), with horizontal magnet interference (b), vertical magnet interference (c), metal board interference (d), and WIFI router interference (e). f. Statical comparison on signal amplitude under different interferences. Square, mean; center line, median; box limits, upper and lower quartiles; whiskers, 1.5×interquartile range; points, amplitudes in response signals; n=40 peak values.

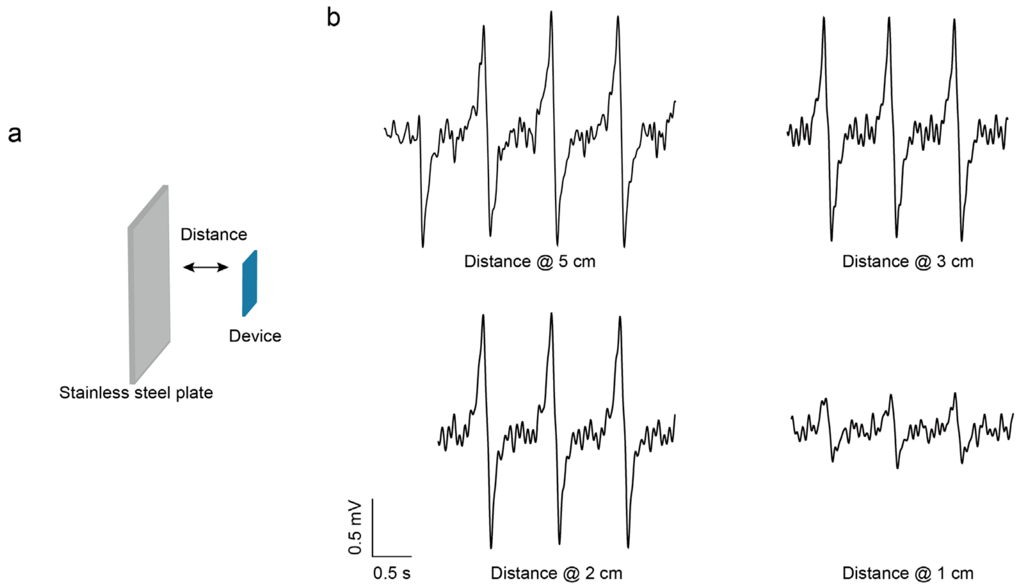

Supplementary Fig. 9 | Anti-interference capability evaluation of the device with interference source distance. a. Schematic illustration on the experimental settings between the interference source and the device. b. Measured response signals under different interference distances.

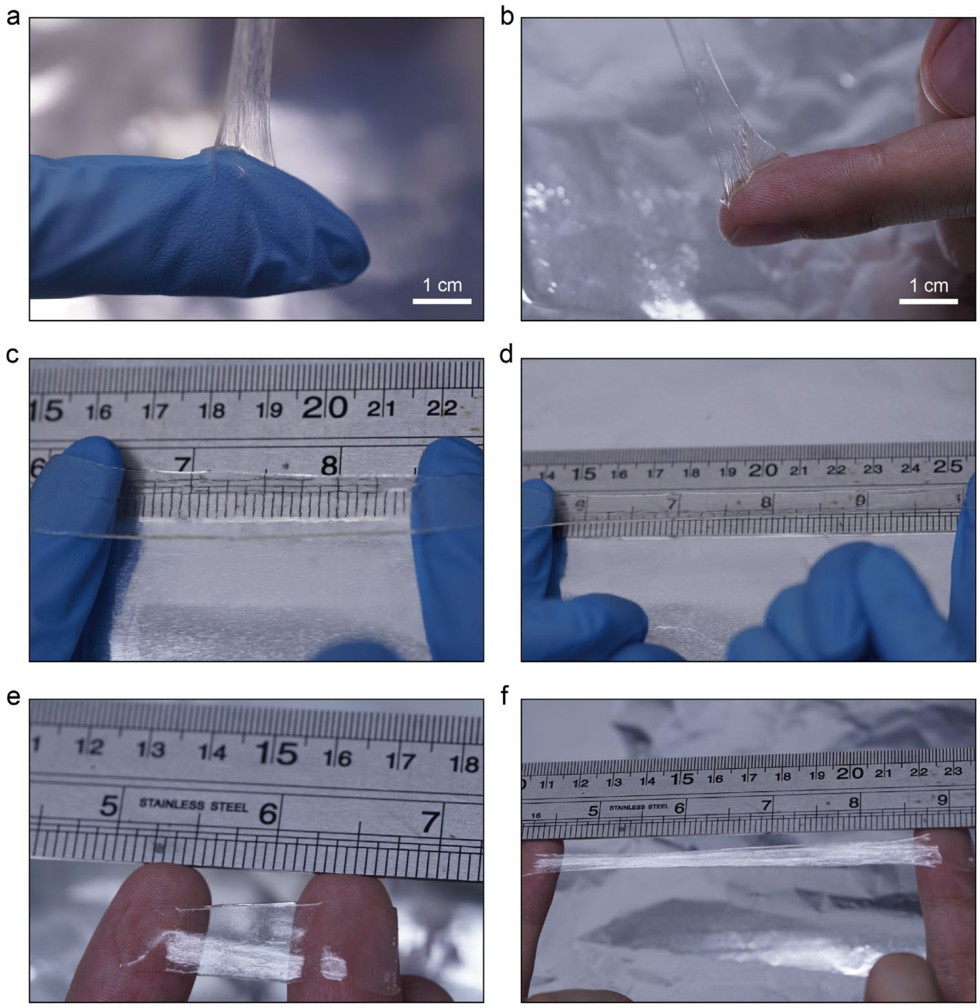

Supplementary Fig. 10 | Optical images showing the strong adhesion of the bio-adhesive with diverse materials. Optical images of the bio-adhesive adhering to nitrile gloves (a) and human skin (b). Optical images showing the original state (c) and stretched over 100% (d) when adhering to nitrile gloves. Optical images showing the original state (e) and stretched over 100% (f) when adhering to human skin.

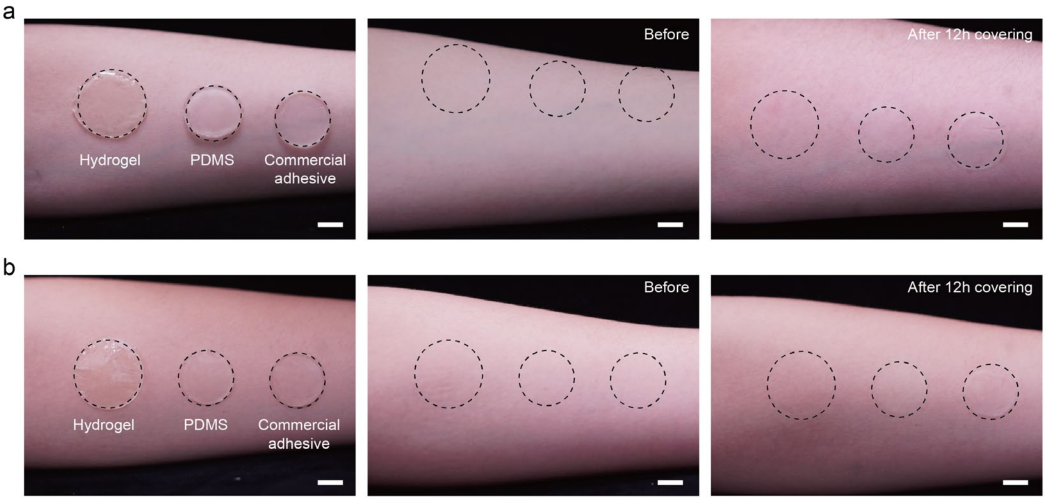

Supplementary Fig. 11 | Biocompatibility of the developed bio-adhesive. a-b. Optical images of two volunteer's forearms taken before and after being covered 12 hours with hydrogel, PDMS, and commercial double-sided adhesive.

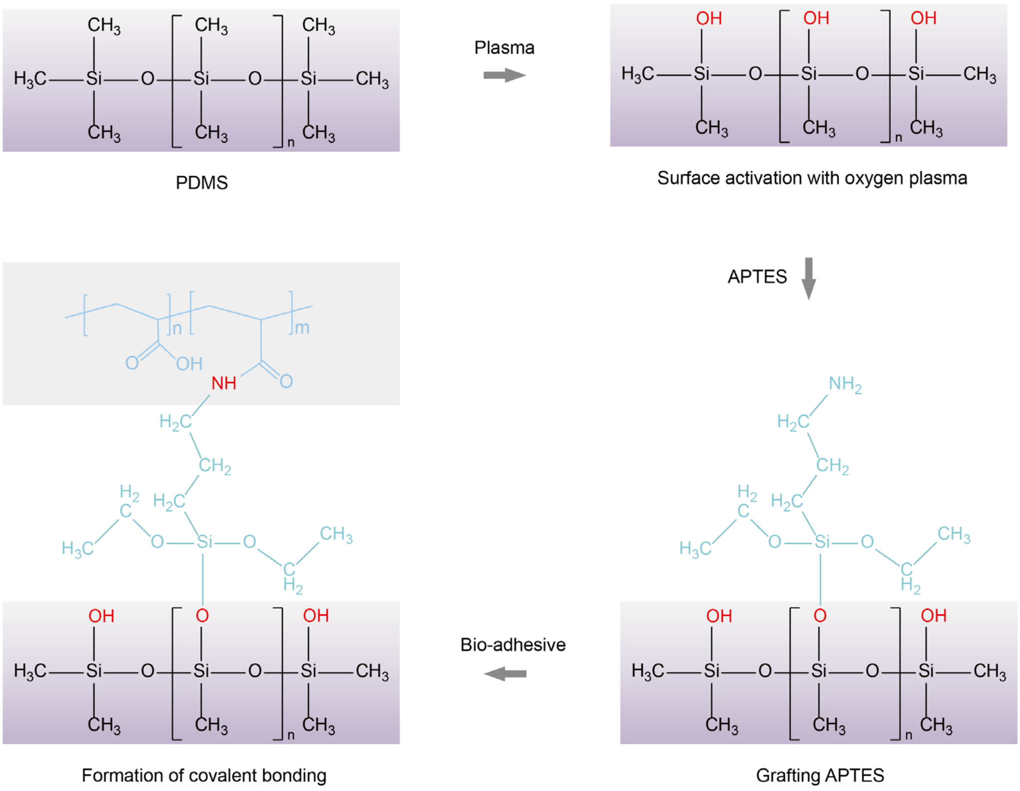

Supplementary Fig. 12 | Preparation process of the amine grafted PDMS.

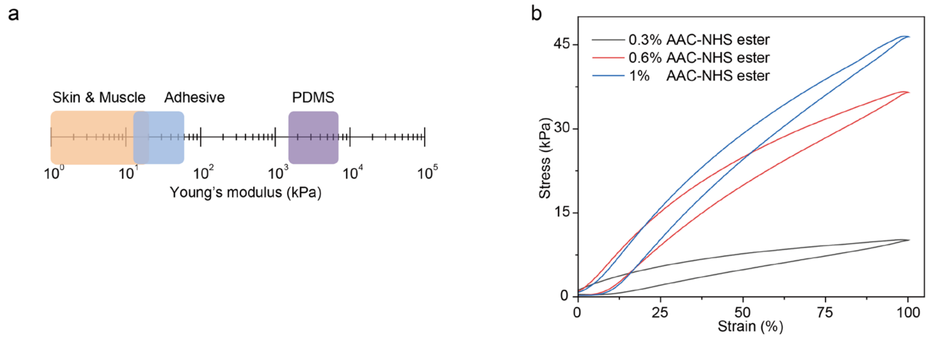

Supplementary Fig. 13 | Mechanical properties of the bio-adhesive. a. Elastic modulus range of human skin, silicone elastomer (PDMS), and the prepared bio-adhesive. The bio-adhesive provides a mechanical match between the human skin and the PDMS. b. Stress-strain curves of the bio-adhesive with AAC-NHS ester content ranging from 0.3% to 1%.

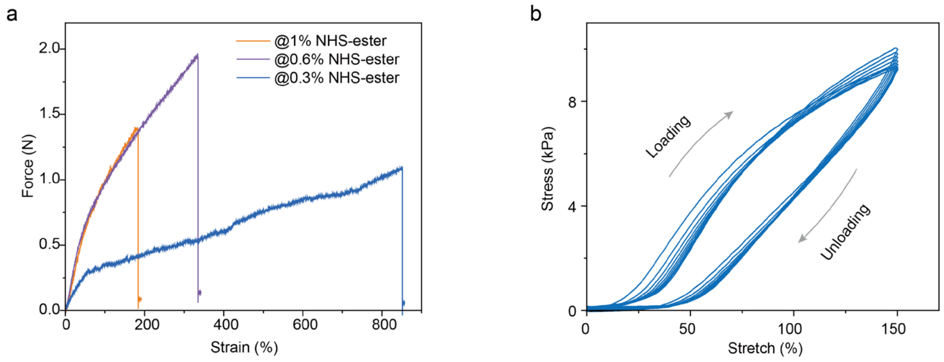

Supplementary Fig. 14 | Mechanical strength and stability of the bio-adhesive. a. Mechanical tests showing the tensile strength of the bio-adhesive with the content of NHS ester ranging from 0.3% to 1%. b. Cyclic test showing the loading and unloading process of the bio-adhesive with the content of NHS ester at 0.3% for maximum strength of 150%.

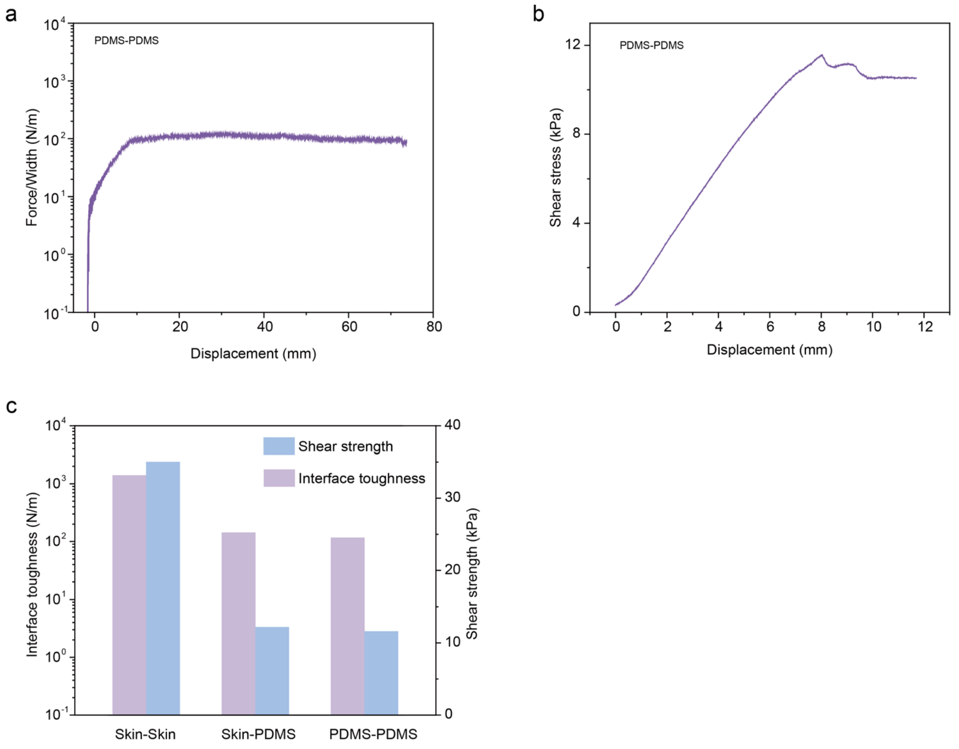

Supplementary Fig. 15 | Interface adhesion performance of the bio-adhesive. a. Force variation and shear stress variation (b) to increased separation distance. c. Interface toughness and shear strength comparison of proposed hydrogel in different interface materials.

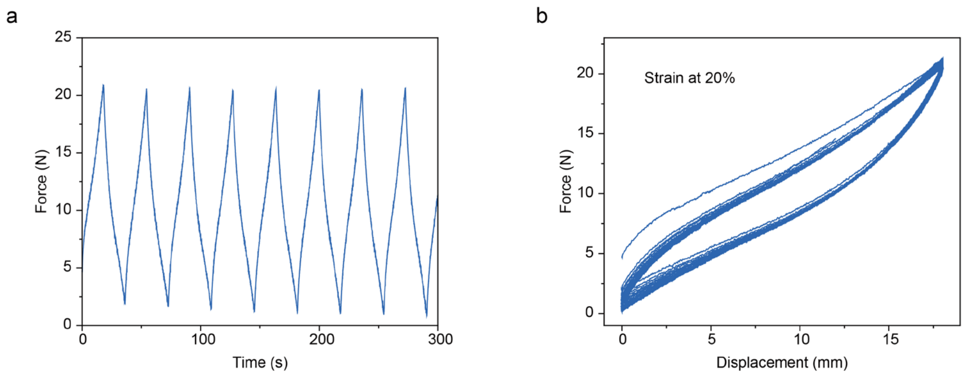

Supplementary Fig. 16 | Cyclic tests of the shear strength with the bio-adhesive as the interface material. a. Cyclic force variation during the test. b. Interfacial stability during a constant strain of 20% for over 50 cycles.

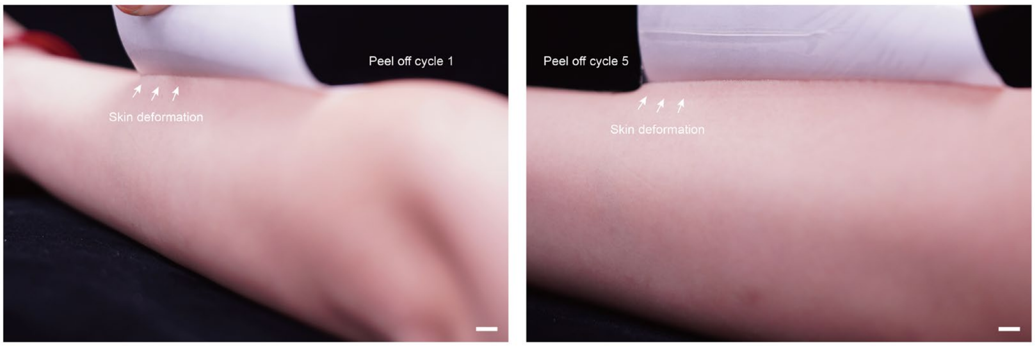

Supplementary Fig. 17 | Optical images of the bio-adhesive after multi peel-off cycles. The skin deformation indicates the bio-adhesive maintained satisfied interface adhesion strength after 5 peel-off cycles. Scale bars: 1 cm.

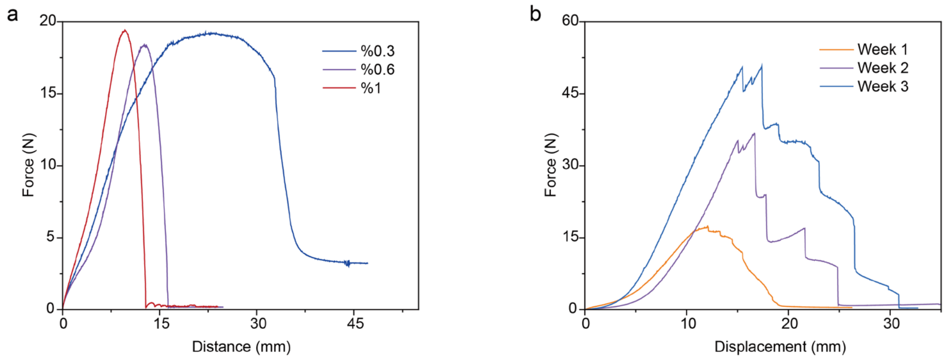

Supplementary Fig. 18 | Shear strength stability of the bio-adhesive after a period of storage. a. Shear strength results of the bio-adhesive after 24 hours storage in room temperature. b. Shear strength results of the bio-adhesive after 3 weeks storage in -20° environment.

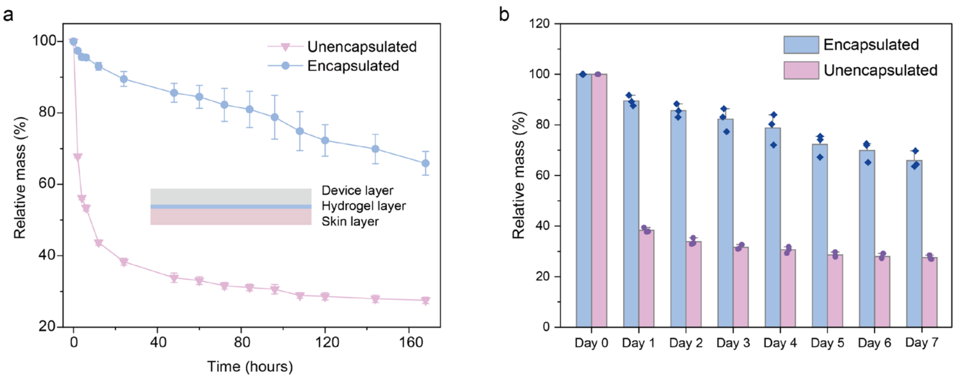

Supplementary Fig. 19 | Anti dehydration performance of the bio-adhesive. a. Weight loss comparison on the bio-adhesive under unencapsulated state and encapsulated by device and skin layer for 7 days in 37℃ environments. b. Statical comparison on the relative mass variation of two groups of hydrogel during 7 days. Bar height, mean; error bars, s.d.; n=3 independent samples.

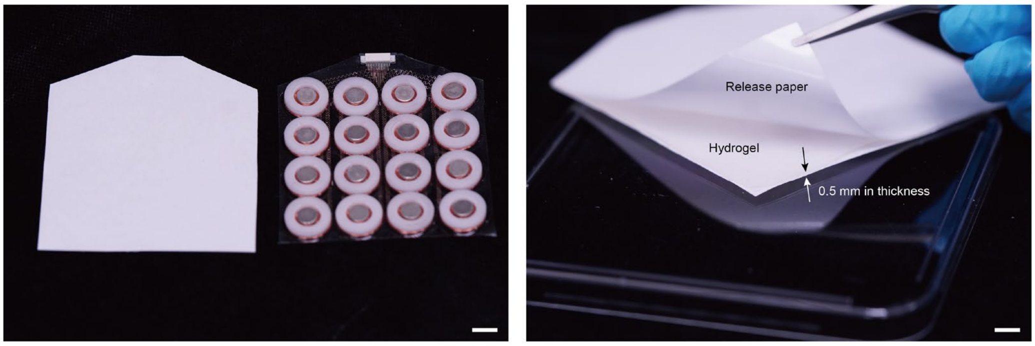

Supplementary Fig. 20 | Optical images of the modular design of the bio-adhesive layer. Scale bars: 1 cm.

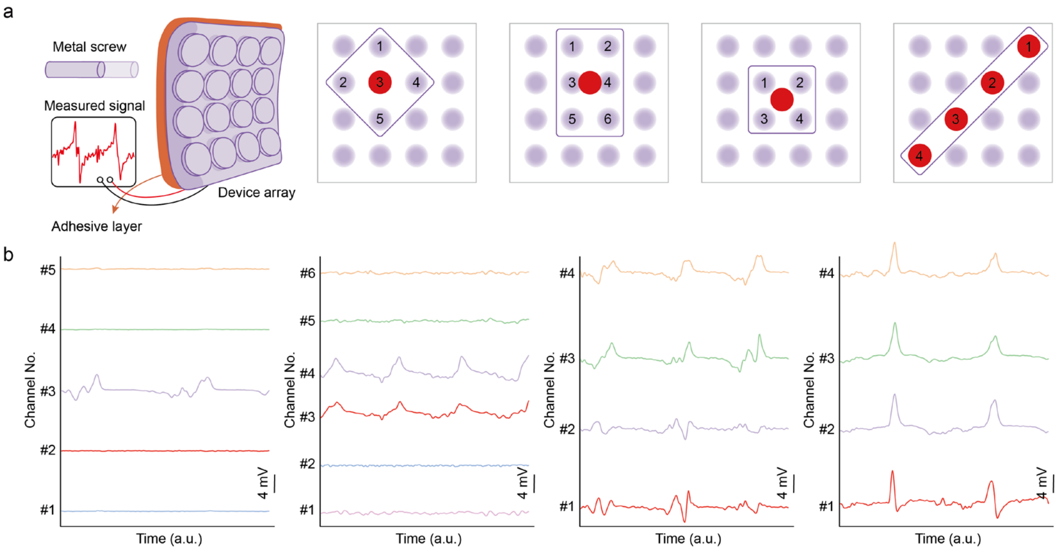

Supplementary Fig. 21 | Test results showing the uniformity and crosstalk suppression capability of BioMDA. a. Schematic illustration of the test patterns. b. Response signal in the sensing units highlighted in (a).

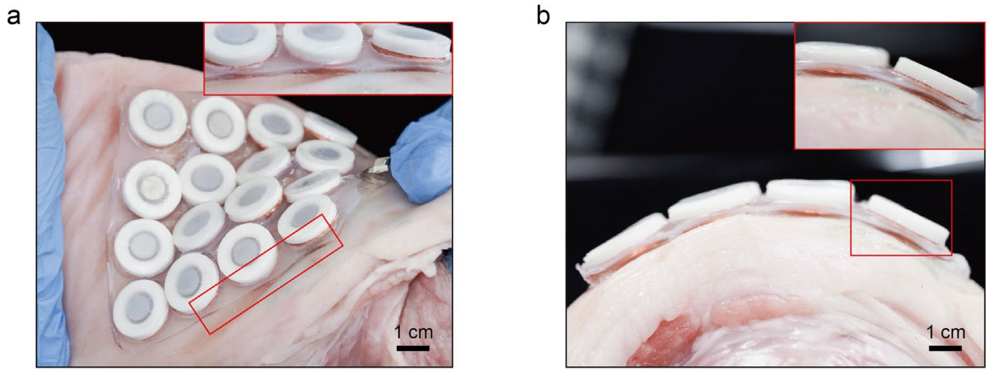

Supplementary Fig. 22 | Optical images show the BioMDA mounted on porcine skin under twisting deformation (a) and bending deformation (b).

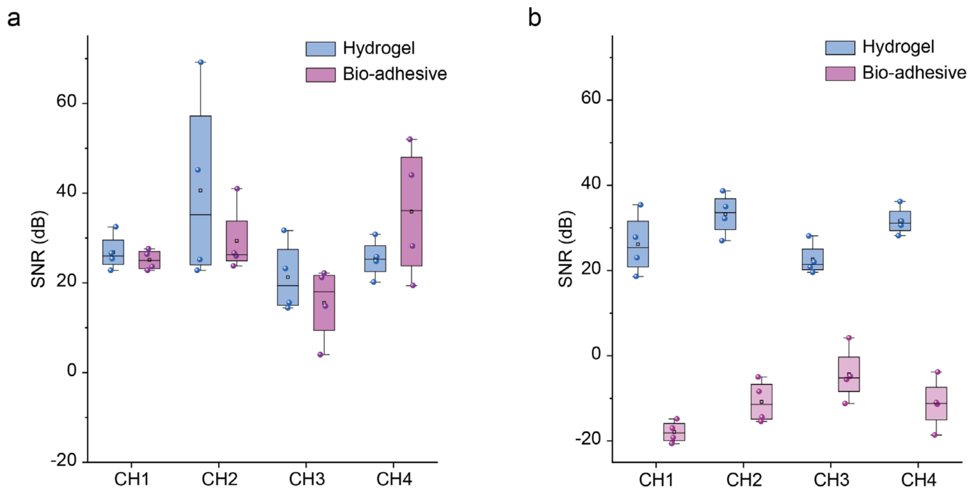

Supplementary Fig. 23 | Signal-to-noise ratio comparison between the bio-adhesive interface and commercial adhesive interface at cycle 1 (a) and cycle 100 (b). Center line, median; box limits, upper and lower quartiles; whiskers, 1.5 × interquartile range; n=4 independent signals.

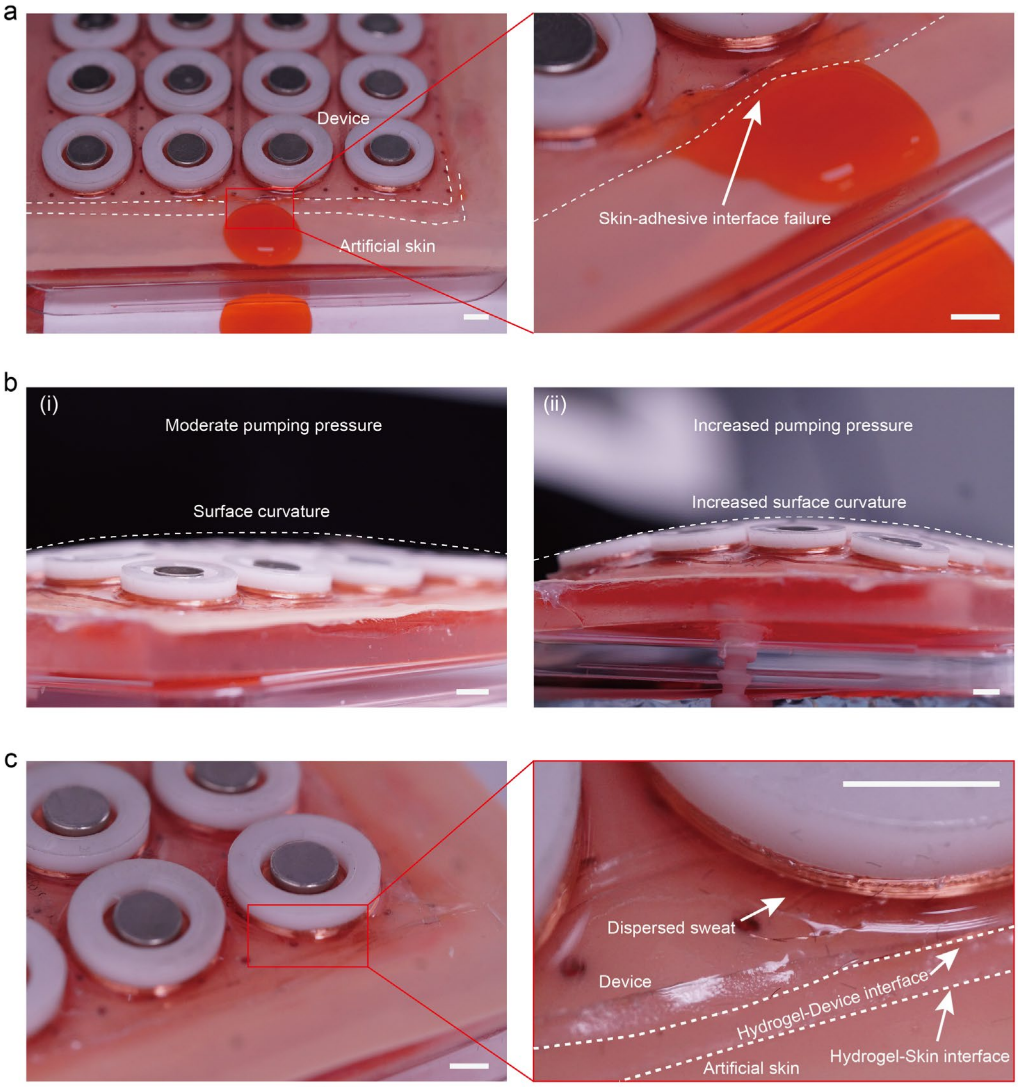

Supplementary Fig. 24 | Interface anti sweat performance comparison between the commercial double-sided adhesive and the bio-adhesive. a. Optical images of the interface adhesion failure with the commercial double-sided adhesive due to sweat accumulation. b. Optical images of the increased surface curvature caused by internal sweat pressure in the bio-adhesive group, proving the seamless interface between the device and the artificial skin. c. Optical images showing the sweat absorption capability of the bio-adhesive under high sweat pressure. Scales bars: 5 mm.

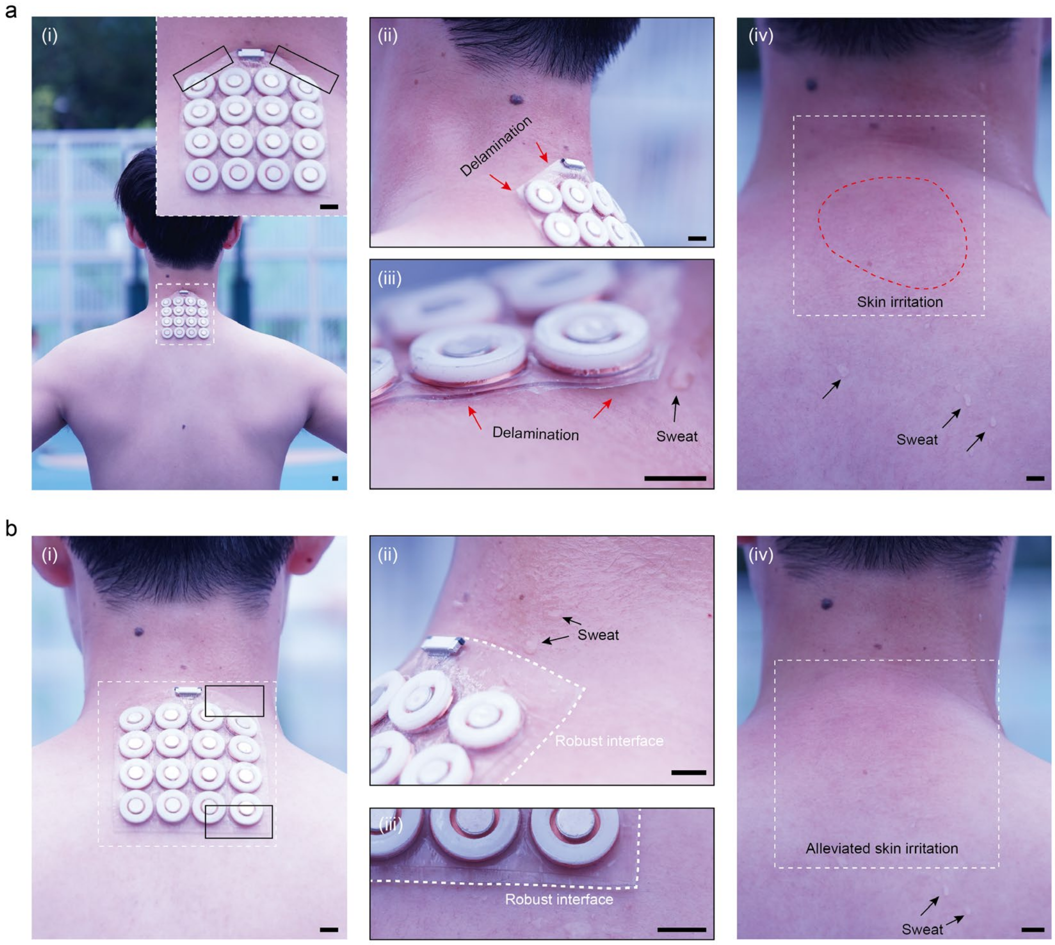

Supplementary Fig. 25 | Interface robustness and biocompatibility comparison between the commercial double-sided adhesive and the bio-adhesive. a. Optical images showing device-skin interface before (i) and after (ii-iv) 30 minutes of intense basketball training. Sweat accumulation and interface delamination were observed after 30 minutes of training. Additionally, akin irritation was also observed due to sweat accumulation on the skin-device interface (iv). b. Optical images showing sweat accumulation and robust device-skin interface in the bio-adhesive group. No sweat irritation was observed in the bio-adhesive group, which can be attributed to the effective sweat absorption reducing sweat accumulation. Scale bars: 1 cm.

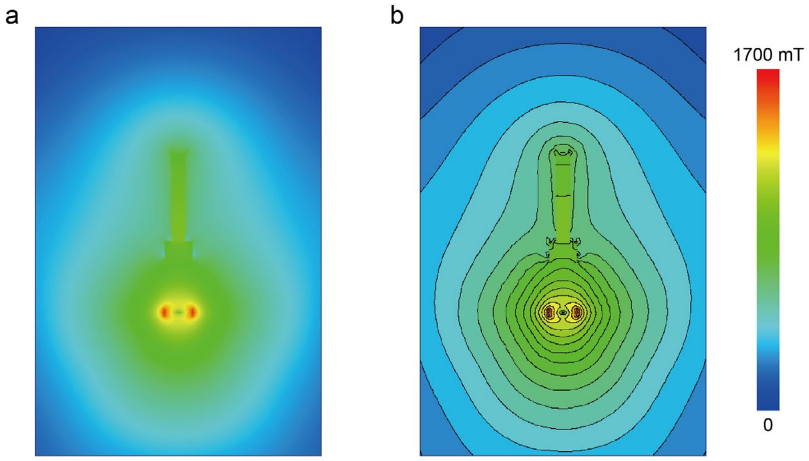

Supplementary Fig. 26 | Simulation results showing the continuous (a) and contour (b) plots of the gradient magnetic field distribution.

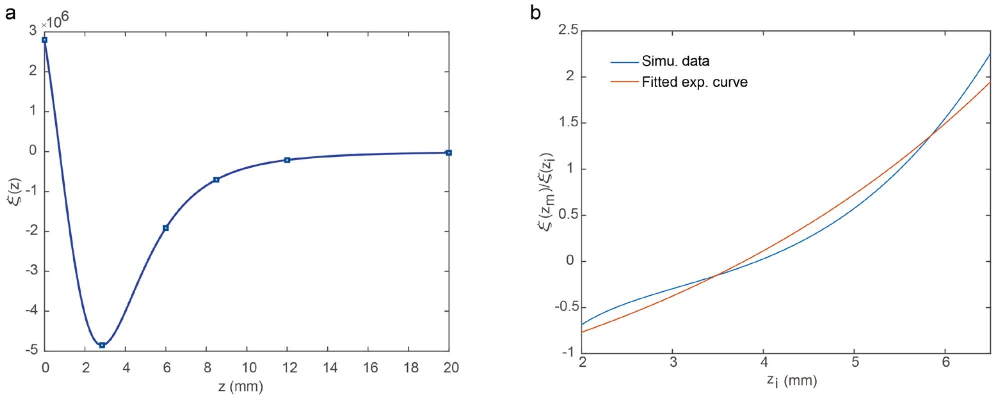

Supplementary Fig. 27 | Simulation of magnet field changes $\xi_B(z)$ for z ranges from 0 to 20mm. a. With a local minimum around 2.834 mm and the scale between the value of $\xi(z_i)$ over $\xi(z_m)$. b. When an implant approaches the sensor from 6 mm away to a minimum distance of 2 mm and the magnet swings up 2 mm.

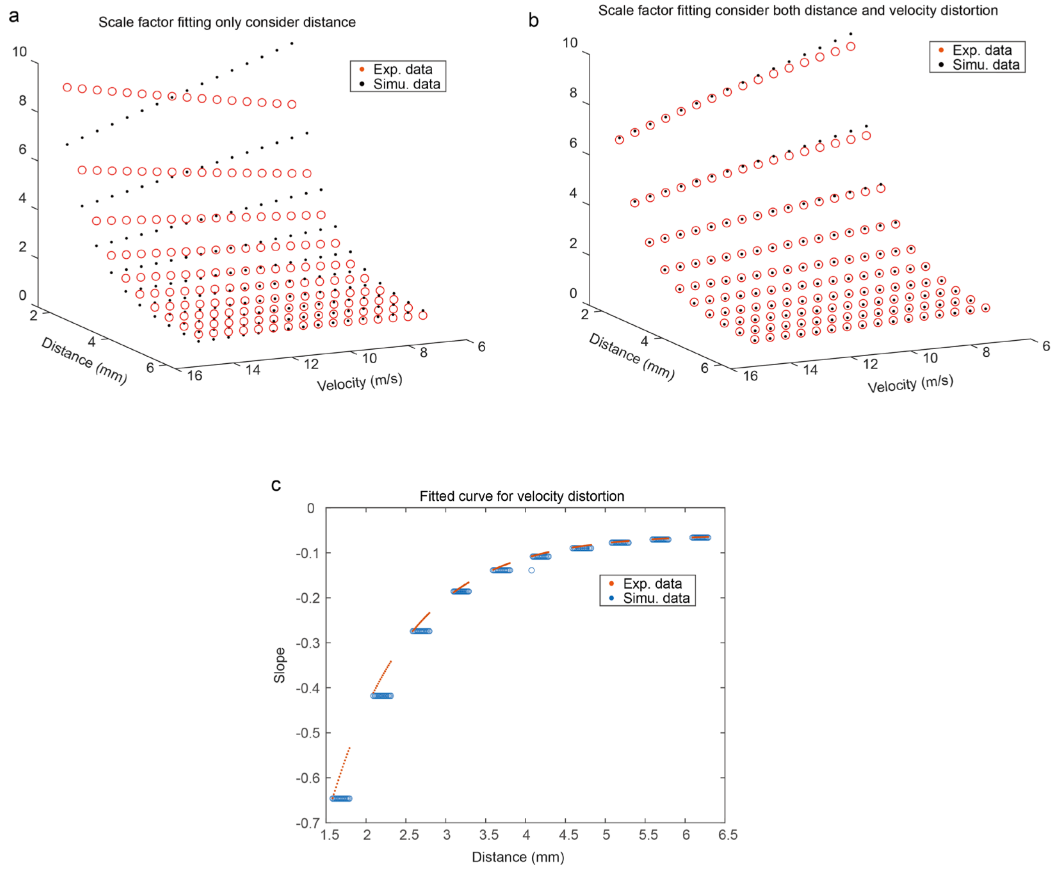

Supplementary Fig. 28 | Comparison between experiment data and simulated model of collectible emf (*ε(z_m)*) by only considering vertical primitives (a), and with both vertical and rotational primitives as $SF(z_i, \dot{z}_i)\xi_B(max(z_i, z_0))\dot{z}_i$ (b), where $SF(z, \dot{z}) = sf_1(z) + sf_2(z)(\dot{z} - (kz + b))$, while the slope values k are plotted in (c).

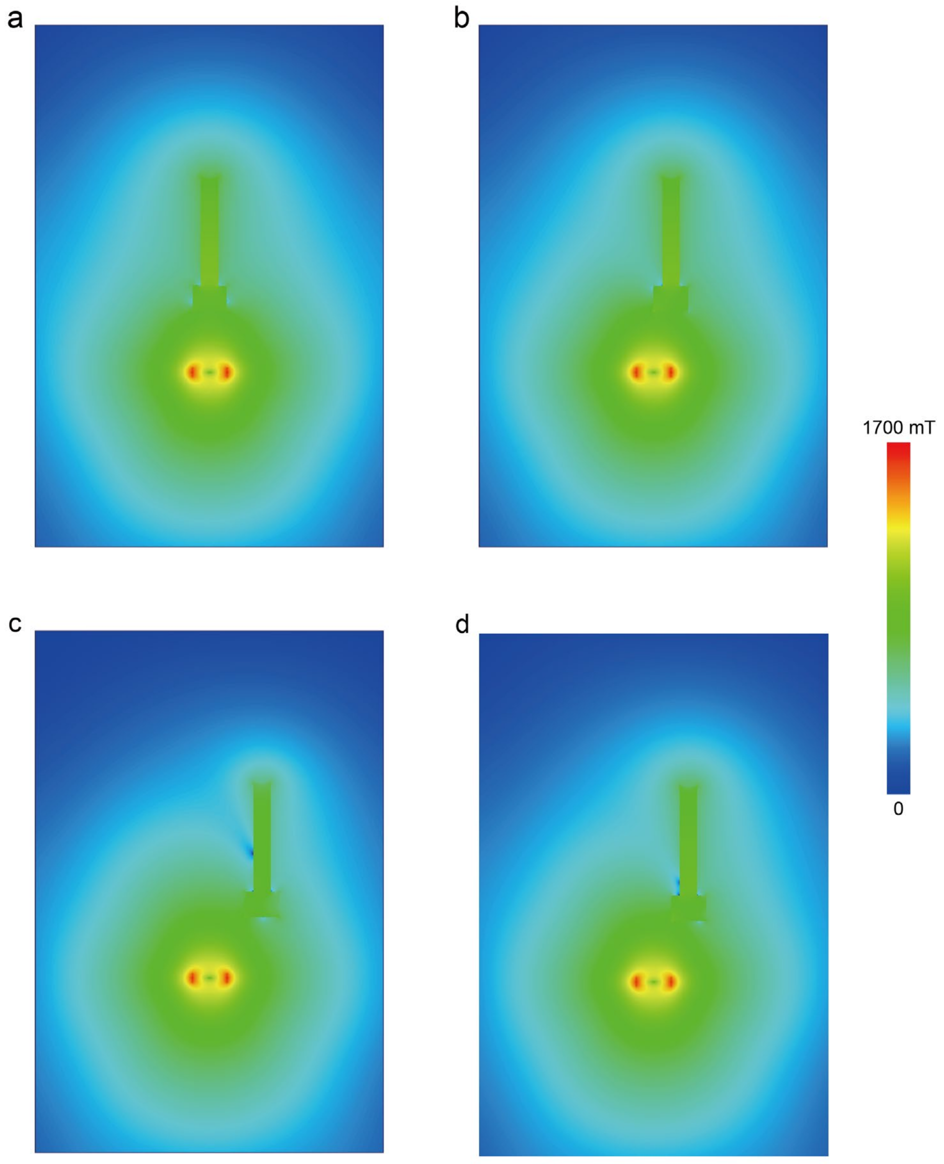

Supplementary Fig. 29 | Simulation results on magnetic field distribution with a set of CPS offsets at 0 (a),4 mm (b), 8 mm (c), and 12 mm (d).

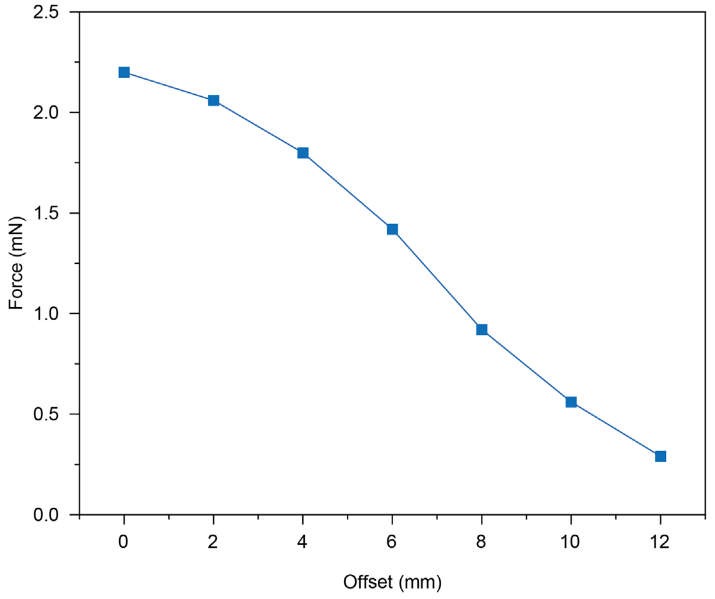

Supplementary Fig. 30 | Inductive force variation with different CPS offsets.

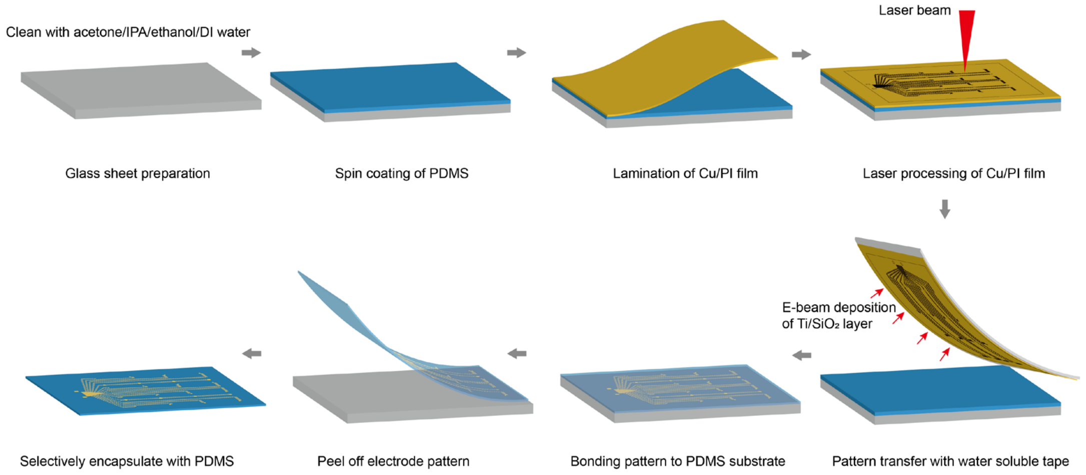

Supplementary Fig. 31 | Fabrication process of the flexible electrode.

References

1. Pooley, R. A. Fundamental Physics of MR Imaging. *RadioGraphics* **25**, 1087–1099 (2005).
2. Edward C. Jordan, K. G. B. *Electromagnetic Waves and Radiating Systems, 2nd edition*. (1968).
3. Prince, J. L. & Links, J. M. *Medical imaging signals and systems*. (Pearson, 2015).
4. Neumann, D. & Kollorz, E. Ultrasound. in *Medical Imaging Systems: An Introductory Guide* (eds. Maier, A., Steidl, S., Christlein, V. & Hornegger, J.) (Springer, 2018).
