# Supplementary material for: Wearable bio-adhesive metal detector array (BioMDA) for spinal implants
Source: Nat Commun. 2024 Sep 6;15:7800. doi: 10.1038/s41467-024-51987-2 (PMC11379874; doi:10.1038/s41467-024-51987-2)
Supplement: Supplementary file 3 — Description Of Additional Supplementary File [file 41467_2024_51987_MOESM3_ESM.pdf]

Description of Additional supplementary files

Supplementary Movie 1: Magnetic field and inductive force variation between the magnet and CPS with decreased vertical distance from 13 mm to 5 mm.

Supplementary Movie 2: Device temperature variation on 1000 cyclic trials.

Supplementary Movie 3: Interface robustness comparison between commercial double-sided adhesive and the bio-adhesive hydrogel.
